# Comparing the effect of a leaflet and a movie in preventing tick bites and Lyme disease in The Netherlands

**DOI:** 10.1186/s12889-016-3146-2

**Published:** 2016-06-10

**Authors:** Desirée Jacqueline Mathieu Angélique Beaujean, Rik Crutzen, Fedor Gassner, Caroline Ameling, Albert Wong, James Everard van Steenbergen, Dirk Ruwaard

**Affiliations:** Centre for Infectiou Disease Control National Institute for Public Health and the Environment, Centre for Infectious Disease Control, P.O. Box 1, 3720 BA Bilthoven, The Netherlands; Department of Health Promotion, Maastricht University, CAPHRI School for Public Health and Primary Care, P.O. Box 616, 6200 MD Maastricht, The Netherlands; Department of Environmental Health, National Institute for Public Health and the Environment, P.O. Box 1, 3720 BA Bilthoven, The Netherlands; Department of Statistics, Informatics, and Mathematical Modeling, National Institute for Public Health and the Environment, P.O. Box 1, 3720 BA Bilthoven, The Netherlands; Leiden University Medical Centre, Centre for Infectious Diseases, P.O. Box 9600, 2300 RC Leiden, The Netherlands; Department of Health Services Research, Maastricht University, CAPHRI School for Public Health and Primary Care, P.O. Box 616, 6200 MD Maastricht, The Netherlands

**Keywords:** Communication interventions, Educational interventions, Ticks, Lyme disease

## Abstract

**Background:**

Lyme disease (LD) has become the most common vector borne illness in the Northern hemisphere. Prevention relies predominantly on fostering protective behaviors (e.g., avoiding tick areas, using protective clothing and repellent, and doing routine tick checks post-exposure). The objective of this study was to evaluate the effectiveness (in terms of knowledge, perceived severity and susceptibility, self-efficacy, response efficacy, intention, and behavior over time) and appreciation of a leaflet and a movie as tools for informing the public in the Netherlands about ticks and LD protective behaviors.

**Methods:**

Participants (1,677 at *t1* and 361 extra at *t2*) were members of a representative Internet panel (adults aged 18 years and above). A four group randomized controlled design was used to test the effect of an information leaflet and a movie (two intervention groups), compared to a control group and a follow-up only control group. Data were collected over two periods: July 15–29, 2013 (*t1*) and at follow-up 4 weeks later, August 16–31, 2013 (*t2*).

**Results:**

Post-intervention results show all respondents in all groups possess good general basic knowledge of ticks and LD. Respondents in both the leaflet and movie groups knew more than respondents in the control group, and had greater awareness of best practices after a tick bite. Intention to perform protective behavior in future was stronger among respondents in the intervention groups. While respondents generally appreciated both the movie and the leaflet, they found the movie ran too long. Follow-up revealed no lasting positive effects from either the leaflet or the movie.

**Conclusions:**

Our results suggest that both the movie and the leaflet are valued and effective intervention tools for improving knowledge about tick bites and strengthening self-efficacy and intentions to perform protective behavior against ticks and LD . Achieving lasting effects, however, calls for more action.

**Electronic supplementary material:**

The online version of this article (doi:10.1186/s12889-016-3146-2) contains supplementary material, which is available to authorized users.

## Background

Lyme disease (LD) is the most common tick borne disease in the United States and Europe. In the Netherlands, where LD is also endemic. The number of general practitioner (GP) consultations for tick bites has increased from 191 per 100,000 in 1994 to 564 per 100,000 in 2009 [1]. In 1994, the incidence of patients visiting the GP for erythema migrans (EM, an associated symptom) was estimated at 39 per 100,000 inhabitants. This number increased to 134 per 100,000 in 2009 [1]. Similar trends have been observed in other European countries [2]. More than one million people in the Netherlands (8 % of the total population) suffered from a tick bite each year, making LD a serious and increasing threat to public health [1, 3]. Sustainable solutions, such as vaccination, are not yet available [4]. Public health intervention therefore relies predominantly on education, focusing on behavioral measures for tick bite prevention (e.g., tick area avoidance, clothing, and repellent use) and Borrelia species transmission prevention post-bite (e.g., checking for ticks and tick-borne pathogen transmission and removing ticks). Despite the availability of public LD- prevention programs, adoption of protective behavior could be improved [5–7]. Beaujean and colleagues found low levels of wearing protective clothing and using repellent in 2013 in the Netherlands. Checking for and removing of ticks were the most adopted measures [8]. Gould, concluded in her study that sustainable LD prevention programs should focus on promoting measures most likely to be adopted. Therefore the Dutch National Institute for Public Health and the Environment (RIVM) focuses in the education materials mainly on 2 measures: checking for and removing of ticks. Since over 10 years the RIVM develops education materials like leaflets and posters on ticks and LD and makes these public available through the internet. Recently the RIVM added an educational movie on ticks and LD to these online materials.

With this study we respond to the call of Mowbray et al. to perform more evaluation studies, since they could find only nine studies in their systematic review that had been undertaken to assess the effectiveness of educational interventions regarding protection against tick-borne disease [9]. This study aims to examine the differences in effectiveness between a leaflet and a movie for several outcomes among the Dutch general public: knowledge, perceived severity and susceptibility, self-efficacy, response efficacy, intention, and behavior over time, as well as subjects’ appreciation of both educational tools. Using study results, we provide advice on how to improve current tick bite and LD health education tools.

## Methods

**Fig. 1:**
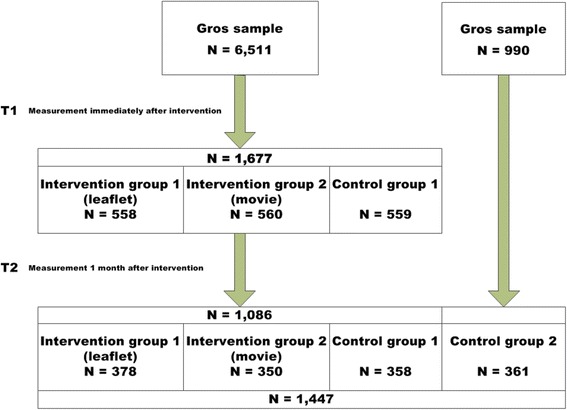
Study design

Our randomized, controlled experiment involved four groups (two intervention groups [IGs] and two control groups [CG]) and two measurement moments (*t1* and *t2*). No prior (pre-test) measurements were taken, but one was done immediately afterwards (*t1*; post-test) and another at 4 week follow-up (*t2*), since both interventions were of brief duration (5 min). At *t1* (July 15–28, 2013), respondents were randomly assigned to three groups (Fig. 1): leaflet group (*IG1*), movie group (*IG2*), and Control group 1 (*C1*)*.* All respondents proceeded to fill out questions on outcome variables. To assess lasting effects of the leaflet and movie, all respondents were asked to participate in *t2* (August 12–31, 2013) 4 weeks after the first measurement. The respondents in the IGs were not re-exposed to the interventions at *t2*. The difference in outcome variables between *t1* and *t2* for each group can be evaluated after adjusting for confounding (see section on statistical analysis below). A difference in group C*1* indicates whether a learning effect is achieved through repeated completion of the questionnaire; this learning effect is assumed to be equally high in all three groups. The null hypothesis (no intervention effect) will therefore hold if differences within intervention groups are equal to that within *C1*. If the difference is greater, the null hypothesis can be rejected in favor of the alternative hypothesis that confirms the presence of an intervention effect.

Outcome variables between *t1* and *t2* may have been influenced by an exogenous factor such as exposure to newspaper articles or television programs on ticks or LD. A new control group (*C2*) was recruited at *t2*, in order to distinguish between the learning effect and exogenous exposure to public information on ticks and LD via mass media or social network or family.

### Participants

Out of a representative Internet panel consisting of 20,000 members set up by Market Response Nederland BV, we invited at *t1* 6,511 members and at *t2* 990 additional persons, to complete the online study. A total of 1,677 respondents participated at *t1* and 1,086 of this group participated at *t2.* From the 990 members invited at *t2*, 361 respondents participated as an extra control group (Fig. 1). Panel members were volunteers drawn from the Dutch general public, willing to participate in online studies. Participants received no reward for study completion. The 20,000 member panel shows a representative distribution of demographic variables (gender, age, region, and level of education) in the Dutch general population. The panel meets high quality requirements and is ISO certified. Participants were invited via email, with a link to the online questionnaire. According to Dutch law [10], this general Internet-based survey involving healthy volunteers from the general population requires no formal medical ethical approval.

### Questionnaire

A questionnaire, developed from a version used in a previous study [8] and expanded to include leaflet and movie appreciation questions, was based on factors derived from the Protection Motivation Theory (PMT) and the Health Belief Model (HBM) [11, 12]. The questionnaire, administered online, began with general questions about activities in green space and personal experiences with tick bites and LD. The groups were then exposed to their allocated intervention or to the control group: IG respondents were presented with the movie or leaflet, while C1 respondents received no information on ticks and LD. In the second questionnaire at *t2* the general questions about activities in green space, personal experiences with tick bites and LD and the questions about the interventions were removed. Moreover, the time frames of the questions about behaviour in the questionnaires differed: the first questionnaire concerned ‘behavior during the last year’, and the same question in the second questionnaire concerned ‘behavior during the last month’. It served to assess level of knowledge (4 questions), perceived severity (6 questions), perceived susceptibility (1 question), self-efficacy (5 questions), efficacy of measures (5 questions), intention (4 questions), behavior (4 questions), appreciation of interventions (5 questions), and activities in green space and experience with tick bites and LD (7 questions) (see Additional file 1 for full questionnaire). All questions, except for true/false knowledge statements, were rated on 7-point Likert scales; responses ranged from “strongly disagree” (1) to “strongly agree” (7).

### Materials

We used the existing paper leaflet created by the National Institute for Public Health and the Environment, and a newly developed online 5-min movie titled *A Tick? Take it!* Both the leaflet and the movie show what ticks look like, where and how they live, how they can make people ill, and how to identify the (early) symptoms of LD. They also explain the reasons, methods, and importance of checking for and removing ticks, when to consult a GP, and how to treat LD. For analogous comparison between the effects of both tools, the leaflet was presented online to ensure any variances in effect would be due to differences in mode rather than offline versus online presentation.

### Statistical analysis

Only respondents who participated in both measurements were included in the analysis (except *C2*, which did not participate at *t1*). Differences in inter-group participant characteristics can still differ despite randomization, and may lead to bias in estimates of effects. This problem was addressed by applying nearest neighbor matching based on propensity scores [13] estimated using random forests, which can outperform logistic regression when the ‘true’ model (i.e., the model from which the data is generated) is highly non-linear [14]. For the propensity score model we choose all variables that we suspect may be correlated with the outcome. We follow the advice by Stuart, by being liberal in our choice of confounders [15]. The loss of efficiency due to the inclusion of too many non-relevant variables is a lesser problem than the exclusion of potentially relevant confounders, as the latter would violate the strongly ignorable treatment assignment assumption that is essential in propensity score matching. Our chosen confounders fall into the following categories. First, basic participant characteristics may affect the outcome variables (age, gender, education,household size, urbanization category for area of residence, geographic region. daily Internet use, and dog or cat ownership); these characteristics were available post-randomization/pre-intervention. Second, characteristics that describe activities or events that are likely to influence the outcomes were also considered. These include frequency of walking/running or mountain biking in green spaces, frequency of gardening, frequency of camping, job requiring work in green spaces, tick bite already contracted by self or anyone in direct social network, LD contracted by self or anyone in direct social network, and previous exposure to the movie or leaflet (for IGs);these variables became available post intervention.

Sum scores for number of correctly answered items were calculated for multiple item questions on knowledge (Q8.1-Q8.7 (7 true/false statements), Q9 (4 preventive measures in 2 situations), and Q11 (6 symptoms) in questionnaire, Tables 1 and 2). Mean (Likert) scores were calculated for all other constructs. Differences in effect size of interventions on knowledge and other constructs between groups were estimated using matched data, applying either a t-test to compare means or a chi-square test to compare proportions (Q10). Intra-group time effect on memorized information was estimated using unmatched data, by applying a paired t-test to compare means or a McNemar test to compare proportions. A multiple testing correction was then performed on all test results using the Benjamini-Hochberg method, to ensure that the overall false discovery rate remained below 5 %. All analyses were performed in R version 3.0.0 or higher, using randomForest for random forest classifier and MatchIt for matching.

**Table 1:** Knowledge questions with correct and incorrect answers

Statements	Answer	
A tick is always bigger than a lady bug	incorrect	
A tick usually falls out of a tree to bite	incorrect	
A tick can be removed by pulling it straight up with a pointed tweezers	correct	
You will always get sick from a tick bite	incorrect	
If you are bitten, it is advisable to remove a tick within 48 h	incorrect	
Ticks always bite on so called ‘hot spots’, such as arm pits, groins, knee cavity	incorrect	
Lyme disease usually starts with a red circle on the skin	correct	
What can you do best?	Situation 1: You will discover a bitten tick on your body, within 24 h after you have been in the green	Situation 2: You will discover a bitten tick on your body, more than 24 h after you have been in the green
Remove tick	correct	correct
Visit general practitioner	incorrect	correct
Note the date and the site of the bite	correct	correct
Monitor your health		
I don’t know	-	-
Questions	Correct answers	Incorrect answers
Imagine you have removed a tick. How long after the bite you should monitor your health?	Up to 3 months after the bite	Up to 3 weeks after the bite Up to 1 year after the bite
Imagine you have removed a tick. On what symptoms you should look?	Red circle on the skin, flu-like, symptoms, painful joints	Bloody nose, diarrhea, hair loss
Imagine you have removed a tick. Which of the symptoms should you watch out for to see if you have got Lyme disease?	Flu-like symptoms	Nose bleed
	Red ring on the skin around the tick bite	Diarrhoea
	Painful joints	Hair loss

**Table 2:** Percentage correct answers on knowledge questions

Number correct answers out of 7 knowledge questions	Control 1 (T1) (*n* = 358)	Control 1 (T2) (*n* = 358)	Control 2 (T2) (*n* = 361)	Movie (T1) (*n* = 350)	Movie (T2) (*n* = 350)	Leaflet (T1) (*n* = 378)	Leaflet (T2) (*n* = 378)
0	0.6	0.6	2.5	0.6	0.3	0.3	0.3
1	1.7	0.3	2.5	0.6	0.3	0	0.3
2	4.7	3.1	6.9	0.9	3.1	2.6	3.4
3	20.1	16.2	17.2	2.6	5.4	6.3	9.8
4	30.2	28.5	31.0	13.1	25.4	18.8	25.1
5	29.9	33.5	29.1	27.1	31.7	32.0	36.8
6	12.0	16.8	10.0	42.9	30.0	25.9	22.0
7	0.8	1.1	0.8	12.3	3.7	14.0	2.4
Number correct answers out of 8 ‘what to do best questions’							
0	0	0.3	0.3	0	0.3	0	0
1	8.9	6.1	8.3	2.9	3.1	2.4	2.6
2	0.3	1.4	1.4	0.9	0.3	0.5	0
3	25.4	18.7	20.5	15.1	16.0	235	15.1
4	5.0	4.5	6.4	3.1	3.1	2.6	4.2
5	14.2	9.5	15.5	8.0	8.0	74	14.3
6	10.9	10.3	11.1	10.9	9.7	13.8	11.6
7	26.3	31.6	26.6	26.6	30.6	25.4	28.0
8	8.9	17.6	10.0	32.6	28.9	24.3	24.1
Recommended health monitoring time							
Correct	26.5	32.1	25.5	69.1	50	74.3	48.4
Number correctly recognized symptoms out of 6 symptoms	Control 1 (T1) (n = 358)	Control 1 (T2) (n = 358)	Control 2 (T2) (n = 361)	Movie (T1) (n = 350)	Movie (T2) (n = 350)	Leaflet (T1) (n = 378)	Leaflet (T2) (n = 378)
0	0	0	0	0	0	0	0
1	0	0	0	0	0	0	0
2	0.3	0.3	0	0	0.3	0	0.3
3	12.3	5.6	13.9	2	4.6	1.3	3.7
4	27.9	21.5	25.8	21.4	22.3	23.3	24.3
5	28.2	31.3	33.5	28.6	36.0	39.9	34.1
6	31.3	41.3	26.9	48.0	36.9	35.4	37.6

## Results

Table 3 shows the baseline characteristics among respondents in all four groups. Although the covariate distributions between groups are largely similar, they do exhibit some differences. For instance, control group 2 has relatively more highly educated respondents, and control group 1 has more respondents from the southern region of the Netherlands. After matching on the propensity score, the balance in covariates between the four groups improved somewhat, in part because the balance was already strong before matching.

**Table 3:** Baseline characteristics in percentages per group

		%	%	%	%
		Control 2 (*n* = 361)	Control 1 (*n* = 358)	Movie (*n* = 350)	Leaflet (*n* = 378)
Gender	Man	48,5	43,3	46	6 43
Age	Woman	51,5	56,7	53,4	56,9
	18-24 years	5,8	5,3	3,4	3,7
	25-34 years	14,1	7,8	8	10,1
	35-44 years	23,5	18,7	26	24,1
	45-54 years	23	26,8	22,3	20,9
	55-64 years	18	24	18,6	19,8
Education	≥65 years	15,5	17,3	21,7	21,4
	High	54,3	42,7	42,9	45,8
	Middle	31,3	35,5	36	36
	Low	14,4	21,8	21,1	18,3
Frequency of walking, running or mountain biking	Least 1 time per week	43,2	39,1	37,7	38,9
	1 to 3 times per month	21,9	19,3	23,4	22
	1 to 11 times per year	22,2	20,7	20,9	23,8
	Never	12,7	20,9	18	15,3
Frequency of gardening	Least 1 time per week	35,5	34,4	41,4	39,7
	1 to 3 times per month	25,2	24,3	20,9	23,8
	1 to 11 times per year	17,2	12,8	15,7	15,6
	Never	22,2	28,5	22	20,9
Frequency of camping	Least 1 time per week	1,4	1,1	0,6	0,5
	1 to 3 times per month	1,1	2	0,6	1,1
	1 to 11 times per year	39,6	27,7	27,4	27,2
	Never	57,9	69,3	71,4	71,2
Are you often actively engaged for your work in green (fe as a ranger or gardener)? Ever had one or more tick bites?	No	96,1	94,7	94,6	96,3
	Yes	3,9	5,3	5,4	3,7
	No	67,6	69	70,9	72,2
	Yes, 1 tick bite	14,1	13,7	13,1	14,8
	Yes, >1 tick bites	13,6	14,2	12	11,1
	Don’t know	4,7	3,1	4	1,9
Has anyone in your immediate environment (such as children, partner, family, friends, colleagues) once one or more tick bites?	No	35,2	37,2	40	37,3
	Yes, in my area it’s happened once	24,4	20,4	22,6	26,5
	Yes, in my area it’s happened more than once	33,2	35,5	27,7	28,8
	Don’t know	7,2	7	9,7	7,4
Have you ever had Lyme disease?	No	96,1	97,5	97,4	98,1
	Yes, I have (had) Lyme Disease (once)	0,8	1,7	2	1,3
	Yes, I have (had) Lyme Disease more than once	0,6	0,3	0,3	0,3
	Don’t know	2,5	0,6	0,3	0,3
	No	69,8	74,6	71,7	75,7
Has anyone in your immediate environment (such as children, partner, family, friends, colleagues) (had) once Lyme disease?	Yes, in my area it’s happened once	19,9	17,9	18,6	18,3
	Yes, in my area it’s happened more than once	5	3,4	5,7	3,2
	Don’t know	5,3	4,2	4	2,9
Did you have the folder / the movie, which you have just read / seen once before read / seen?	Yes	NA	NA	2,3	16,9
	No	NA	NA	93,4	79,6
	Don’t know	NA	NA	4,3	3,4
Family size	1 person	21,3	20,9	23,7	20,4
	2 persons	41,6	41,6	39,7	37,8
	3 persons	13,3	11,5	13,4	17,2
	4 persons	18	15,1	12,6	16,4
	≥5 persons	5,8	10,9	10,6	8,2
Urbanity	Very strong	22,7	18,2	17,4	20,9
	Strong	31	23,7	26,6	24,3
	Moderate	17,5	19	21,1	18,8
	Little	17,7	25,7	21,7	22,8
	Not	11,1	13,4	13,1	13,2
Geographical region	3 biggest municipalities (Amsterdam, Rotterdam, Den Haag)	14,4	12,3	13,1	13
	West (Utrecht, Noord-Holland, Zuid-Holland excl. 3 biggest municipalities)	31,6	21,5	28,3	29,6
	North (Groningen, Friesland, Drenthe)	10,2	8,1	10,9	10,6
	East (Overijssel, Gelderland, Flevoland)	16,1	22,9	20	20,6
	South (Zeeland, Noord-Brabant, Limburg)	22,7	30,7	24,3	22,5
	Border municipalities	5	4,5	3,4	3,7
Owner dog or cat	≥1 dog(s)	15,5	17	15,4	14
	≥1 cat(s)	15,8	15,6	16,9	18,5
	dog(s) and cat(s)	5	4,7	5,7	6,9
	No	63,7	62,6	61,7	60,6
Internet use per week/month	1 days	1,9	0,3	0,6	0,8
	2 days	0,8	2,8	1,1	2,1
	3 days	2,5	4,5	3,1	2,4
	4 days	3	3,9	5,1	4,2
	5 days	8,3	8,4	6,6	8,2
	6 days	6,6	8,9	12,9	10,3
	7 days	74,2	69	68	69,3
	2 to 3 times per month	0,6	0,6	0	0,3
	once per month	0	0,6	0	0
	< once per month	0,3	0,6	0,6	0,3

The sample derived differed slightly in composition (age distribution) from that of the general population: respondents tend to be older than non-respondents. Examination of age stratified results (not presented) shows no systematic influence for any age stratum. We therefore consider the results to be relevant to the general population. In the Appendix additional results are presented in Table 6 the proportions or means (depending on the operationalization of the outcome involved) of all tested outcomes and in Table 7 the differences in group mean between control groups.

### Knowledge

**Fig. 2:**
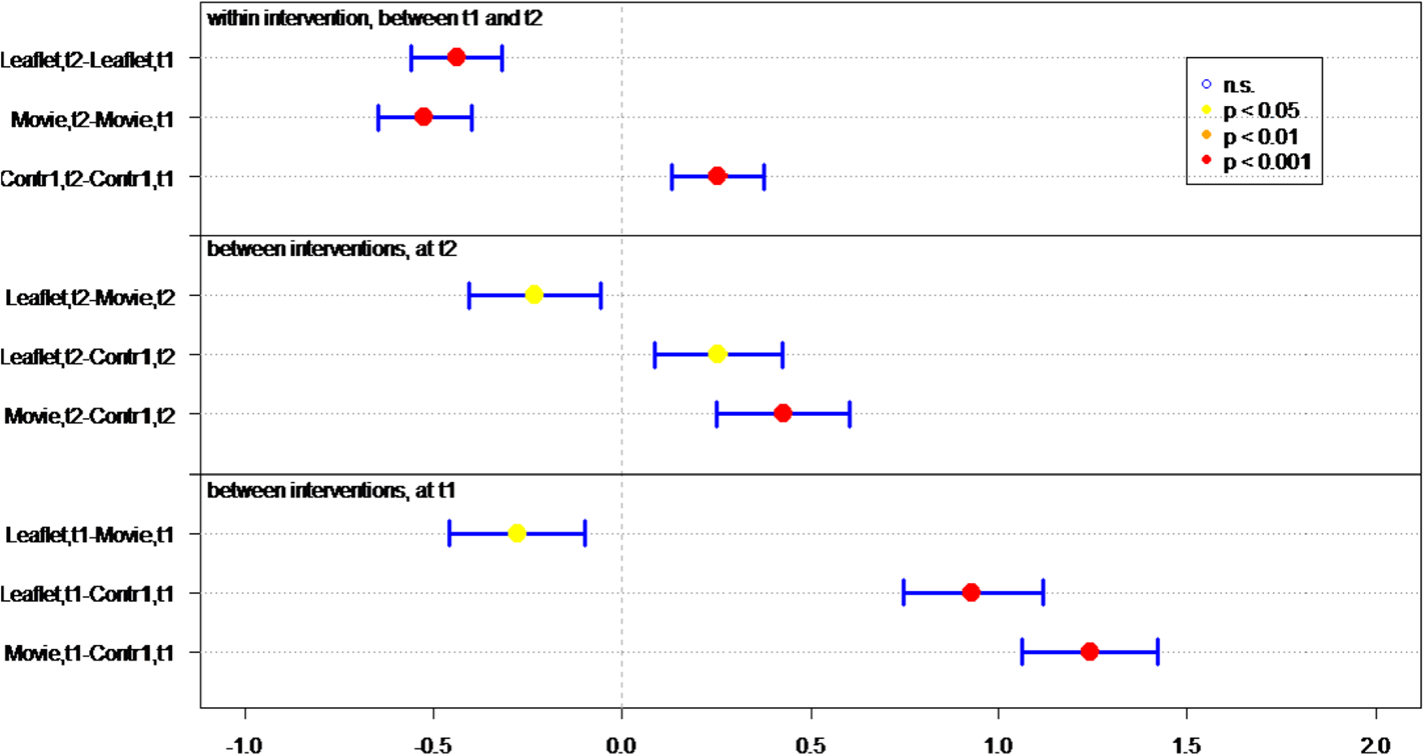
Differences in mean sumscores on knowledge per group (leaflet, movie or control) on *t1* (immediately after intervention) and *t2* (after 1 month)

Of all respondents (IGs and controls), 83.8 % answered at least 4 out of 7 knowledge statements (Table 2) correctly. Knowledge sum scores for respondents who viewed the movie or received the leaflet were significantly better than for controls, at both *t1* (immediately post-intervention) (*P* < 0.01 and *P* < 0.01, respectively) and *t2* (4 weeks post-intervention) (*P* < 0.01, *P* = 0.02, respectively) (Fig. 2). Respondents who viewed the movie had a significantly better knowledge sum score on both measurements (*P* = 0.01 at t1, *P* = 0.03 at t2), compared to respondents who received the leaflet. Respondents in both IGs had lower knowledge scores at *t2* than at *t1* (for both *P* = <0.01).

**Fig. 3:**
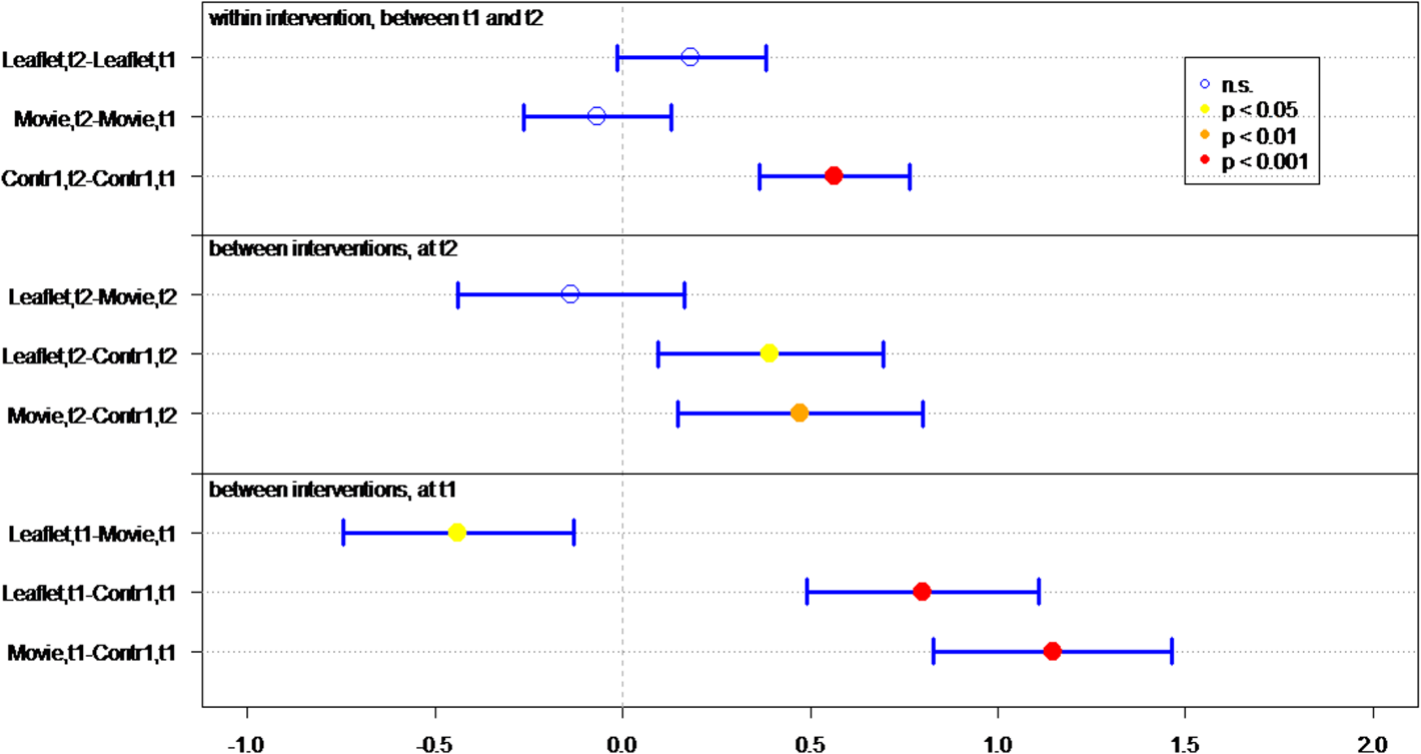
Differences in mean sumscores on knowledge about behavior in case of tick bite per group (leaflet, movie or control) on *t1* (immediately after intervention) and *t2* (after 1 month)

Respondents’ knowledge of optimal measures in both situations (discovery of tick bite on body *within* or *more than* 24 h after time spent in green space) was significantly better in both IGs than in the control groups, at both *t1* (both *P* < 0.01) and *t2 (*movie *P <* 0.01, leaflet *P =* 0.05*)* (Table 2 and Fig. 3).

**Fig. 4:**
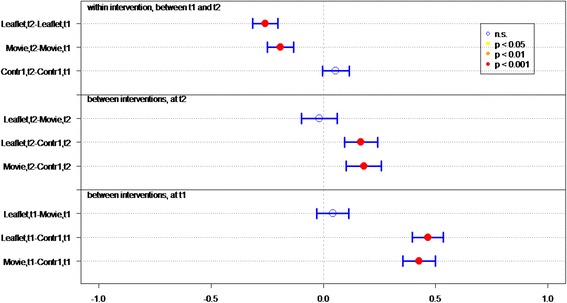
Differences in knowledge about health monitoring time per group (leaflet, movie or control) on *t1* (immediately after intervention) and *t2* (after 1 month)

Respondents who viewed the movie or received the leaflet had significantly better knowledge of how long they should monitor their health after a tick bite, on both measurements, than did respondents in control groups (both *P* < 0.01) (Table 2 and Fig. 4).

**Fig. 5:**
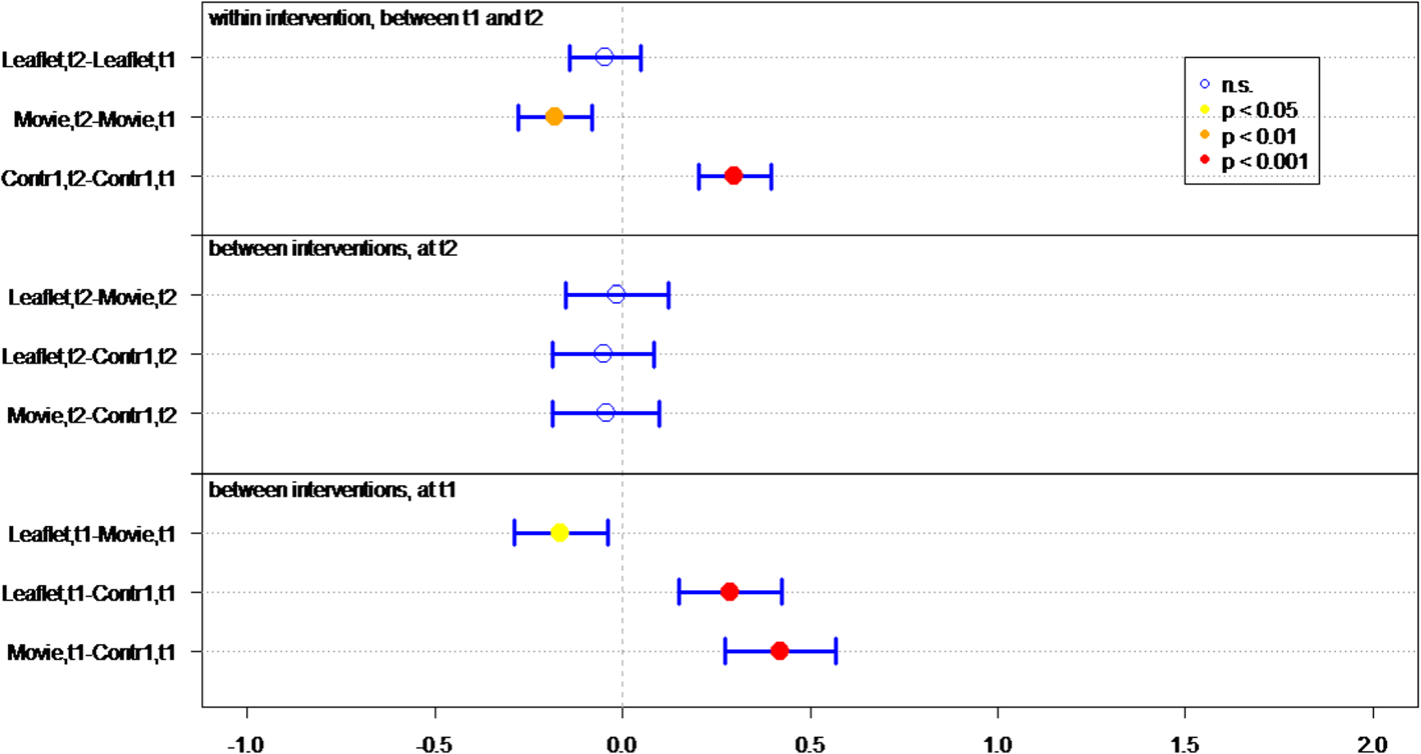
Differences in recognizing symptoms per group (leaflet, movie or control) on *t1* (immediately after intervention) and *t2* (after 1 month)

Initial LD symptoms (red circle on skin, flu-like symptoms, and painful joints) were significantly better known by IG than control group respondents at *t1* (both *P* < 0.01) (Table 2 and Fig. 5). Respondents who viewed the movie had significantly more knowledge of symptoms than did respondents in the leaflet group (*P* < 0.05, Fig. 5). Additionally, more IG respondents could identify at least one symptom of LD, compared to the control groups.

### Perceived anxiety, susceptibility and seriousness

No significant differences in perceived anxiety and perceived susceptibility were found, with respect to tick bites and LD, between any groups. The respondents perceived LD on average as a very serious disease (M = 6.15). For not significant results see additional file 2.

### Self-efficacy and response efficacy

**Fig. 6:**
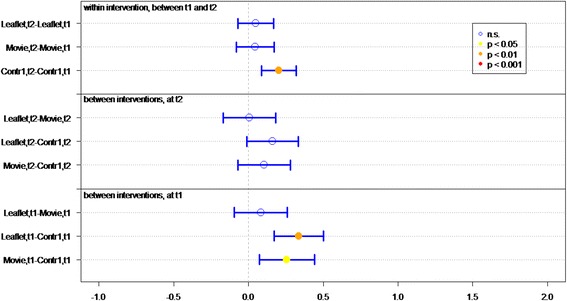
Differences in mean Likert scores on self efficacy per group (leaflet, movie or control) on *t1* (immediately after intervention) and *t2* (after 1 month)

The self-efficacy and response efficacy of respondents (both IG and control group respondents) is high (Table 4); the mean score for self- efficacy in the IGs was 5.42 and 5.28 in the control groups (scale 1–7). At *t1*, the self-efficacy of IG respondents is higher than of control group respondents (movie *P* = 0.02, leaflet *P* < 0.01, Fig. 6*)*.

**Table 4:** Mean Likert-scores of self-efficacy and response efficacy of measures (answer scales: 1 = strongly disagree - 7 = strongly agree)

	C2	C1, T1	C1, T2	Movie, T1	Movie, T2	Leaflet, T1	Leaflet, T2
I manage to recognize a tick on my body	5.49	5.43	5.62	5.59	5.77	5.52	5.69
I manage to do a tick check after every visit to the green	4.52	4.62	4.72	4.77	4.76	4.79	4.87
I manage to use a pointed tweezers (or any other kind of tick remover) to remove a tick	5.04	5.03	5.26	5.47	5.36	5.47	5.4
I manage to record the place and the date of the tick bite	5.5	5.39	5.66	5.82	5.82	5.81	5.85
I manage to visit the GP^a^ if a tick is more than 24 h in the skin	5.66	5.53	5.75	5.74	5.91	5.97	5.99
Do you think recognition of a tick helps to prevent LD^b^?	5.57	5.71	5.75	5.84	5.79	6	5.88
Do you think tick check after each visit to the green helps to prevent LD^b^?	5. 9	5.7	5.66	5.73	5.65	5.93	5.83
Do you think removing immediately a tick with a pointed tweezers helps to prevent LD^b^?	5.19	5.36	5.42	5.69	5.54	5.83	5.65
Do you think recording the place and date of the tick bite helps to prevent LD^b^?	3.83	4.01	4.27	4.31	4.3	4.43	4.37
Do you think visiting the GP^a^ with a tick more than 24 h in the skin helps to prevent LD^b^?	4.75	5	5.03	4.93	5.06	5.25	5.26

^a^GP general practitioner, ^b^LD Lyme Disease

Taken together, all respondents scored highest (M = 5.79) on seeing a GP within 24 h if a tick had bitten into their skin and least high (M = 4.72) on self-efficacy for performing a tick check after every visit to a possibly tick infested area.

**Fig. 7:**
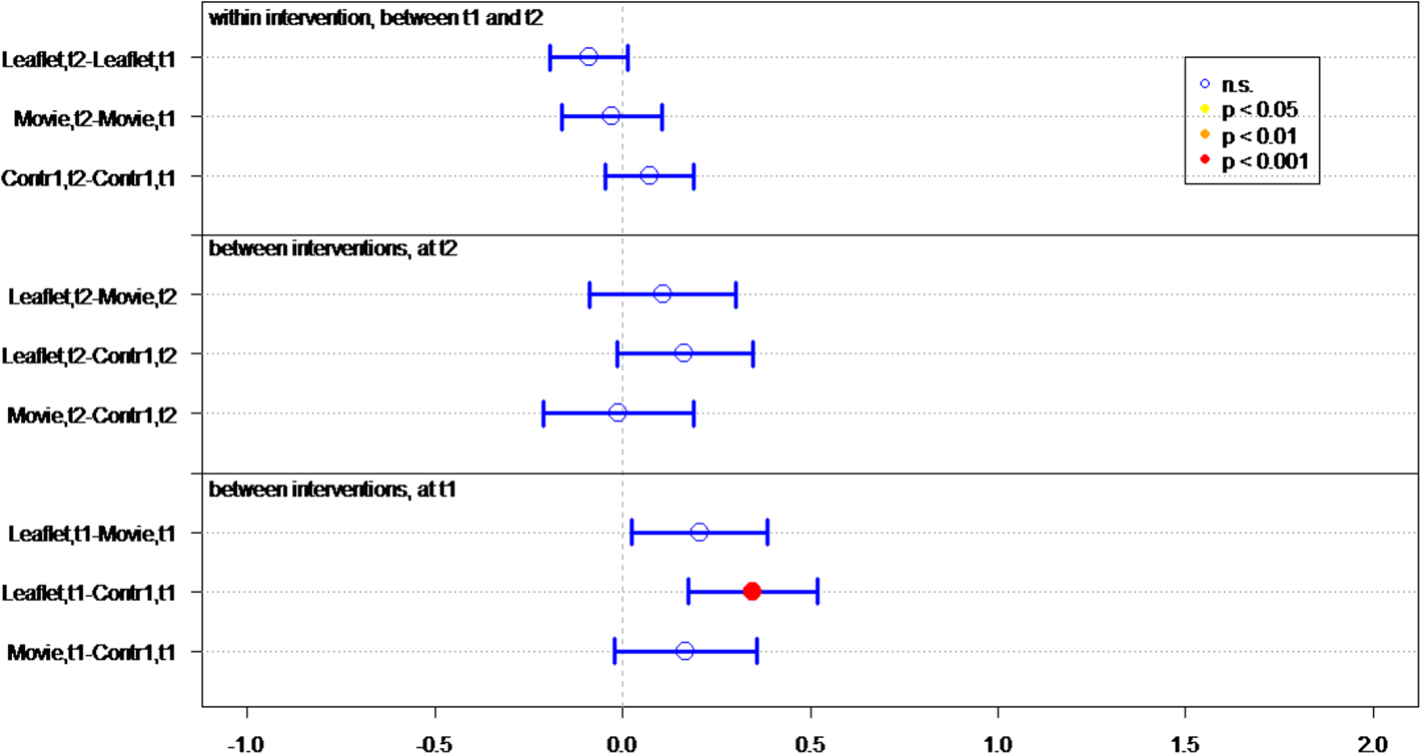
Differences in mean Likert scores on perceived efficacy per group (leaflet, movie or control) on *t1* (immediately after intervention) and *t2* (after 1 month)

The response efficacy of respondents in the leaflet group is significantly higher (*P* < 0.01), at *t1*, than of the control group respondents (Fig. 7). In general, all respondents rated “recognition of a tick” as the most effective measure for preventing LD (M = 5.79), and “recording the place and date of the tick bite” as the least effective measure (M = 4.22).

### Intention

**Fig. 8:**
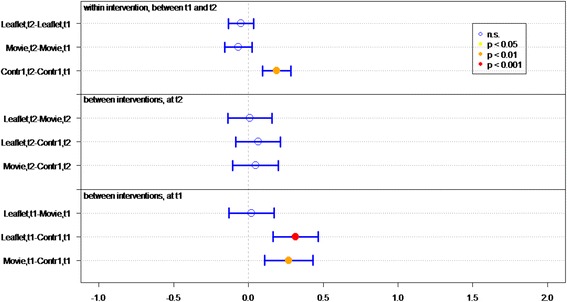
Differences in mean Likert scores on intention per group (leaflet, movie or control) on *t1* (immediately after intervention) and *t2* (after 1 month)

At *t1*, significantly more IG respondents (*P* < 0.01) expressed the intention of immediately removing a tick after discovery, recording place and date of tick bite, and visiting a GP if the tick remained on the skin for more than 24 h, compared to control group respondents (Fig. 8).

### Behaviors

At *t1*, the protective behavior of the respondents (such as checking on ticks, tick removal, recording date and place of tick bite, and visiting a GP) was the same in all groups.

### Lasting effects

At *t2*, 1 month after *t1*, the sum score on knowledge and the question “How long after the bite should you monitor your health?” decreased significantly in both IGs (Figs. 2 and 4). Furthermore the knowledge on symptoms decreased significantly in the movie group (Fig. 5).

About 20 % of respondents has reported a tick bite themselves or to someone else during the month between *t1* and *t2*. These respondents visited nature more often than respondents who had not reported a tick bite between *t1* and *t2* (99 % versus 80 %). Respondents in IGs who reported a tick bite at *t1* as well as at *t2,* reported more often to take some tick bite prevention measures (like recording of date and place of a tick bite (movie and leafet group) and tick checks (movie group) at *t2* (question concerned performed behavior during last month) than at *t1* (question concerned behavior performed during last year). But they reported less often to take other tick bite prevention measures (like immediate tick removal (movie and leaflet group) and tick checks (leaflet group) (Table 5).

**Table 5:** Mean Likert scores of taking preventive measures of respondents who reported a tick bite at *t1 and t2* (answer scale 1–7)

Preventive measures	Control 1 (t1) (*n* = 48)	Control 1 (t2) (*n* = 48)	Movie (t1) (*n* = 39)	Movie (t2) (*n* = 39)	Leaflet (t1) (*n* = 46)	Leaflet (t2) (*n* = 46)
Tick check	4,31	4,44	3,49	4,15	4,83	4,18
Tick removal	4,23	4,6	4,13	3,77	4,61	4,52
Note date and site of bite	2,91	3,47	3,11	3,2	2,8	2,83
Visit general practitioner	1,97	1,55	1,81	1,81	2,19	1,6

### Appreciation

The appreciation of the interventions differed only on the length of time spent. Respondents who received the leaflet were significantly (*P* < 0.01) more satisfied with length of time spent, compared to respondents who viewed the movie.

### Effect of questionnaire

Repeated completion of the questionnaire had a positive effect on knowledge and intention. Respondents in *C1*, who filled out the questionnaire twice, achieved a significant better knowledge sum score (*P* < 0.001, Fig. 2), knew significantly better what to do if a tick bite is discovered on the body (*P* < 0.001, Fig. 3) – both within and after 24 h of time spent in green space – recognized significant more LD symptoms at *t2* than at *t1* and had significant more frequently the intention to perform protective behavior (*P* < 0.001, Fig. 5). This underlines the assigned effect of repeatedly filling in the questionnaire, notwithstanding the effect of public exposure to information on ticks and LD *(C2)*.

## Discussion

The aim of the current study was to evaluate and compare the effects of a newly developed movie and a leaflet for preventing tick bites and LD. We conclude that all respondents in this representative sample of the Dutch population possessed solid knowledge of ticks and LD at baseline: more than 80 % answered at least 4 out of 7 knowledge statements correctly. This may be the positive effect of the annual nationwide “Week of the Tick” campaign, launched over a decade ago. Nonetheless, overall knowledge scores in both IGs increased significantly, compared to the control group. This is comparable with the results of Lawless and colleagues, who described that knowledge increased significantly in the group who received an instructional video [16]. This finding is relevant since others have demonstrated that increased knowledge seems to positively influence protective behavior [17]. However, research in LD-endemic areas has shown that, despite adequate knowledge of LD symptoms and transmission, many people have not adopted behaviors to reduce their infection risk [18, 19]. These findings suggest that (like many other protective health behaviors, ranging from washing hands to using condoms to stop STI transmission) lack of knowledge is only one reason for poor uptake of protective behavior.

The self-efficacy of IG-respondents was higher versus the control group respondents and also the response efficacy in the leaflet group . This is an important and promising effect of the leaflet and the movie because self-efficacy and response efficacy are essential determinants in taking appropriate actions. Floyd showed in his meta-analysis of the literature on the PMT that coping variables like self-efficacy and response efficacy were strongly related to intention and behavior [20, 21]. Other researchers also conclude in their studies that ‘a belief’ in the response efficacy and in the own ability to perform the measures is the primary goal of sustainable LD and tick prevention programs [17, 18, 22, 23].

Significantly more respondents (both IGs) also expressed the intention of taking more easily implementable preventive measures, e.g., immediately removing a tick, recording place and date of tick bite, and visiting the GP if the tick remained on the skin more than 24 h post-bite. This effect is promising, since many interventions only have effect on knowledge and attitudes [9].

Finally, none of both interventions had a lasting positive effect on knowledge after 1 month. Lasting effects of the interventions on behavior were not analysed statiscally because the time periods differed too much (1 year versus 1 month)*.*. The RIVM is currently assessing the effect of our “Tick bite” mobile app, which contains extra information and features that complement the leaflet and movie. Repeated use of this app may lead to lasting effects in behavior changes.

The main comment on the movie was the length (5 min). We have already addressed this by splitting the movie in two short parts (1.5 min): one part is about checking for ticks and one about removing ticks.

The second control group was added in order to analyze the effect of repeated questionnaire completion and distinguish it from public exposure to information on ticks and LD via mass media. We found an assigned effect for repeated questionnaire completion, notwithstanding the effect of public exposure to information on ticks and LD.

The present study has some limitations. The respondents are members of an online panel. This implies that all respondents in this study have internet access. Since internet access is extremely high (97 %) in the Netherlands, we assume that this will not adversely affect the representativeness of this study http://statline.cbs.nl/Statweb/publication/?DM=SLNL&PA=71098ned&D1=33-133&D2=0-2&D3=a&VW=T. Furthermore, members of an online panel are probably a certain selection of people, but the respondents are not selected on the fact that they are interested in ticks and LD. So it is not plausible that this will reduce the representativeness of the results. As usual in web-based research, we used data based on self-reported health risk behaviors. This is often associated with social desirability. Crutzen et al. demonstrated that three longitudinal studies revealed no meaningful associations between social desirability and self-reported health risk behaviors in web-based research [24]. Hence, we view the Internet as an appropriate medium to collect self-reports on health risk behaviors.

Knowingly, no prior (pre-test) measurements were taken because another measurement pre-intervention would entail respondents filling out the questionnaire twice in quick succession. Viewing the interventions while able to remember the pre-test measurement questions, which may have influenced their post-test measurement answers (e.g., due to recall or assessment reactivity).

Respondents at *t2* were slightly older than non-respondents. However, a sensitivity analysis suggests that indications and the significance of this study’s intervention effects are unlikely to change with a fully representative age distribution, although treatment effects may differ slightly in actual magnitude.

## Conclusion

Despite the limitations, we conclude that respondents appreciate the leaflet and movie, and both interventions increased knowledge, self-efficacy, and intention with regard to ticks and LD. While the movie is more effective in furthering knowledge, the leaflet is better for increasing the response efficacy. Since there were no lasting effects of interventions on behavior measured, repeated exposure to them, or additional intervention efforts, are needed. Both online intervention tools present two advantages over written ones: they are available anywhere and anytime, and repeated exposure is easier to achieve. This information is still only available online, however, and the public would have to visit the website and find the movie and the leaflet themselves.

## Appendix

**Table 6:** The means or proportions of all tested outcomes

Outcomes	Control2 t2	Control1 t1	Control1 t2	Movie t1	Movie t2	Leaflet t1	Leaflet t2	Overall
Sum scores on knowledge	4.03	4.2	4.45	5.41	4.89	5.13	4.69	4.69
Sum scores on behavior in case of tick bite	5.08	4.98	5.54	6.14	6.07	5.77	5.95	5.65
Fraction of correct answers on health monitoring time	0.25	0.27	0.32	0.69	0.5	0.74	0.48	0.47
Sum scores on recognizing symptoms	4.73	4.78	5.08	5.23	5.05	5.1	5.05	5
Likert scores on perceived severity overall	4.45	4.51	4.49	4.51	4.53	4.46	4.39	4.48
Likert scores on anxiety	3.87	3.87	3.84	3.89	3.9	3.76	3.69	3.83
Likert scores on seriousness	5.03	5.15	5.14	5.14	5.15	5.17	5.1	5.12
Likert scores on susceptibility	2.89	3.04	2.96	3.03	2.88	3.03	2.92	2.96
Likert scores on self-efficacy	5.24	5.2	5.4	5.48	5.52	5.51	5.56	5.42
Likert scores on perceived efficacy	4.95	5.16	5.23	5.3	5.27	5.49	5.4	5.26
Likert scores on intention	5.56	5.66	5.85	5.96	5.9	5.97	5.92	5.83

**Table 7:** Test results for differences in group mean between Control group 1 at *T1* and Control group 2 at *T2*

Outcome	Difference (Cont1,*T1*–Contr2, *T2*)	Confidence Interval	*P*-value
Sum scores on knowledge	0.202	(-0.001 - 0.405)	0.08
Sum scores on behavior in case of tick bite	−0.016	(-0.353 - 0.321)	0.88
Sum scores on recognizing symptoms	0.045	(-0.117 - 0.207)	0.80
Likert scores on perceived severity overall	0.034	(-0.148 - 0.216)	0.88
Likert scores on anxiety	−0.033	(-0.281 - 0.215)	0.87
Likert scores on seriousness	0.101	(-0.075 - 0.278)	0.43
Likert scores on susceptibility	0.186	(-0.027 - 0.398)	0.32
Likert scores on self-efficacy	0.024	(-0.152 - 0.201)	0.98
Likert scores on perceived efficacy	0.237	(0.043 - 0.432)	0.06
Likert scores on intention	0.089	(-0.077 - 0.255)	0.52
Fraction of correct answers on health monitoring time	0.019	(-0.053 - 0.091)	0.89

## Additional files

Additional file 1: Questionaire (Questions 1 – 7 were excluded in the measurements at *t2*. Question 7 was excluded for the controlgroups). (DOCX 32 kb) Additional file 2: Additional figures not significant results. (DOCX 133 kb)
