# Supplementary material for: Comparing the effect of a leaflet and a movie in preventing tick bites and Lyme disease in The Netherlands
Source: BMC Public Health. 2016 Jun 10;16:495. doi: 10.1186/s12889-016-3146-2 (PMC4902941; doi:10.1186/s12889-016-3146-2)
Supplement: Additional file 1: — Questionaire (Questions 1 – 7 were excluded in the measurements at t2. Question 7 was excluded for the controlgroups). (DOCX 32 kb) [file 12889_2016_3146_MOESM1_ESM.docx]

**Questionaire (Questions 1 – 7 were excluded in the measurements at *t2*. Question 7 was excluded for the controlgroups)**

Introduction

Welcome to the Study group
The following questions are about ticks and Lyme disease.

It is important to us that you answer all the questions, even if you sometimes find them difficult and that some questions are similar to each other. Cross the answer that is most true for you. There are no ‘right’ or ‘wrong’ answers. This is about your answers and your experiences. For this reason we ask that you answer the questions yourself without looking at the internet or asking anyone else what they think.

The outcomes will be treated in strict confidence, processed anonymously and will be used in this study only. Participation is voluntary and you may stop at any time without giving a reason.

We wish you every success in answering these questions and thank you in advance for your time and trouble.

Please use the buttons on the screen and not the browser buttons. Please answer the question on the screen by clicking on the box in front of the answer. Filling in the questionnaire takes about 10 minutes.

1. How often to you take part in the activities below?

*When we talk about green spaces we mean being outdoors in nature areas such as woods, heathland, dunes or a park.*

|  | Minimum of once a week | 1-3 times a month | 1-11 times a year | Never |
| --- | --- | --- | --- | --- |
| Walking, running or mountain biking in green spaces |  |  |  |  |
| Gardening |  |  |  |  |
| Camping |  |  |  |  |

2. Does your job involve you actively working in green spaces (e.g. forester or gardener)?

*When we talk about green spaces we mean being outdoors in nature areas such as woods, heathland, dunes or a park.*

- No
- Yes

3. Have you ever had one or more tick bites?

- No
- Yes, I have had one tick bite
- Yes, I have had more than one tick bite
- I can’t remember

4. Has anyone in your direct social network (such as children, partner, family, friends, colleagues) ever had one or more tick bites?

- No
- Yes, it has happened once in my social network
- Yes, it has happened a few times in my social network
- I can’t remember

5. Have you ever had Lyme disease?

- No
- Yes, I have Lyme disease, or have had it once
- Yes, I have had Lyme disease more than once
- I can’t remember

6. Has anyone in your direct social network (i.e. children, partner, family, friends, colleagues) ever had Lyme disease?

- No
- Yes, it has happened once in my social network
- Yes, it has happened a few times in my social network
- I can’t remember

We are now going to show you a film about tick bites. When the film has finished a button will appear. Click on it to go to the next question. You can start the film by clicking on it. The film lasts for 5 minutes.

Or:

We are now going to show you a leaflet about tick bites. Please read the leaflet very carefully. It can be read by scrolling downwards. You can only move on to the next question when the ‘next’ button appears at the bottom of the screen. This takes about 3 minutes.

Or:

We are now going to show you a leaflet about tick bites. Please read the leaflet very carefully. It can be read by scrolling downwards. You can only move on to the next question when the ‘next’ button appears at the bottom of the screen. This takes about 3 minutes.

7. Have you ever seen/read the film/leaflet you have just seen/read before?

- yes
- no
- I can’t remember

8. You are now going to see a number of statements. Please read them and indicate if you think the statement is ‘true’ or ‘false’. If you do not know the answer please mark ‘I don’t know’.

|  | true | false | I don’t know |
| --- | --- | --- | --- |
| A tick is always larger than a ladybird. |  |  |  |
| A tick usually falls from a tree in order to bite. |  |  |  |
| Your can remove a tick by pulling it directly upwards with pointed tweezers. |  |  |  |
| A tick bite always makes you ill. |  |  |  |
| If you are bitten it is advised to remove the tick within 48 hours. |  |  |  |
| Ticks only bite in so-called warm areas, e.g. in the armpit, groin or behind the knee. |  |  |  |
| In Lyme disease a red ring usually appears on the skin. |  |  |  |

9. Mark the box that indicates the best course of action to take in the following situations. Please do this for Situation 1 and Situation 2. These are followed by a number of actions. Please mark what you think are the best course/s of action. It is possible to mark more than one box.

*(More than one answer is possible)*

|  | Situation 1. You find a tick attached to your body within 24 hours of being in an area infested by ticks. | Situation 2. You find a tick attached to your body more than 24 hours after being in an area possibly infested by ticks. |
| --- | --- | --- |
| Remove the tick |  |  |
| Go to your general practitioner (GP) |  |  |
| Note down the date and place of the bite in your diary |  |  |
| Monitor your health |  |  |
| I don’t know |  |  |

10. Imagine you have removed a tick. For how long after you have been bitten should you monitor your health?

- up to 3 weeks after the bite
- up to 3 months after the bite
- up to 1 year after the bite
- I don’t know

11. Imagine you have removed a tick. Which of the symptoms below should you watch out for to see if you have got Lyme disease?

*(More than one answer is possible)*

- Nose bleed
- Flu-like symptoms
- Painful joints
- Diarrhoea
- Red ring on the skin around the tick bite
- Hair loss
- I don’t know

12. Please indicate to what extent you agree or disagree with the statements below?

|  | 1 – strongly disagree | 2 | 3 | 4 -  neutral | 5 | 6 | 7 – strongly agree |
| --- | --- | --- | --- | --- | --- | --- | --- |
| I am frightened of a tick bite |  |  |  |  |  |  |  |
| I am frightened of getting a red ring on my skin. |  |  |  |  |  |  |  |
| I am frightened of Lyme disease. |  |  |  |  |  |  |  |
| If you get a tick bite, this is serious. |  |  |  |  |  |  |  |
| If you get a red ring on your skin after a tick bite, this is serious. |  |  |  |  |  |  |  |
| If you get Lyme disease, this is serious. |  |  |  |  |  |  |  |

13. In your opinion, how big is the risk that you will be bitten by a tick in the coming year?

*If you think the risk is very high then answer a 7. If you think the risk is very low then answer a 1. Of course you can answer any number between 1 and 7.*

- 1 – very low
- 2
- 3
- 4 - neutral
- 5
- 6
- 7 – very high

14. In your opinion, what is the risk that you will get Lyme disease in the coming year?

*If you think the risk is very high then answer a 7. If you think the risk is very low then answer a 1. Of course you can answer any number between 1 and 7.*

- 1 – very small
- 2
- 3
- 4 - neutral
- 5
- 6
- 7 - very high

15. Imagine that you are going to take one of the precautions below to prevent getting Lyme disease. From a practical point of view, to what extent do you think that you would really succeed in taking these precautions?

*When we talk about green spaces we mean being outdoors in nature areas such as woods, heathland, dunes or a park.*

|  | 1 – strongly disagree | 2 | 3 | 4 -neutral | 5 | 6 | 7 – strongly agree |
| --- | --- | --- | --- | --- | --- | --- | --- |
| I would be able to recognise a tick on my body. |  |  |  |  |  |  |  |
| I would be able to check my body for tick bites after every trip to a green space. |  |  |  |  |  |  |  |
| I would be able to remove a tick immediately with pointed tweezers (or other type of tick remover). |  |  |  |  |  |  |  |
| I would be able to note down the place of the tick bite on my body and the date in my diary. |  |  |  |  |  |  |  |
| I would be able to go to the GP if I had a tick on my skin for over 24 hours. |  |  |  |  |  |  |  |

16. The following questions concern measures to prevent Lyme disease. Please indicate how much you think that these measures would help?

*When we talk about green spaces we mean being outdoors in nature areas such as woods, heathland, dunes or a park.*

|  | Certainly does not help | 2 | 3 | 4 -neutral | 5 | 6 | 7 – certainly does help |
| --- | --- | --- | --- | --- | --- | --- | --- |
| Do you think that recognising a tick when checking the body helps to prevent Lyme disease? |  |  |  |  |  |  |  |
| Do you think that checking for ticks after every visit to a green space helps to prevent Lyme disease? |  |  |  |  |  |  |  |
| Do you think that removing a tick immediately with pointed tweezers or other type of tick remover helps to prevent Lyme disease? |  |  |  |  |  |  |  |
| Do you think that noting down the place and date of the tick bite helps to prevent Lyme disease? |  |  |  |  |  |  |  |
| Do you think that going to the GP if you have had a tick on your skin for longer than 24 hours helps to prevent Lyme disease? |  |  |  |  |  |  |  |

17. The following questions concern the way the people around you deal with tick bites and Lyme disease.

*When we talk about green spaces we mean being outdoors in nature areas such as woods, heathland, dunes or a park.*

|  | 1 – strongly disagree | 2 | 3 | 4 -neutral | 5 | 6 | 7 – strongly agree | I don’t know |
| --- | --- | --- | --- | --- | --- | --- | --- | --- |
| People who are important to me check their body after every visit to a green space to prevent Lyme disease. |  |  |  |  |  |  |  |  |
| People who are important to me think it is important that I check my body for ticks after every visit to a green space. |  |  |  |  |  |  |  |  |
| People who are important to me would remove a tick immediately after they discovered it on their body. |  |  |  |  |  |  |  |  |
| People who are important to me think it is important that I remove a tick as soon I find it on my body. |  |  |  |  |  |  |  |  |
| People who are important to me would note down the place of the tick bite on their body and the date. |  |  |  |  |  |  |  |  |
| People who are important to me think it important that I note down the place of the tick bite on my body and the date. |  |  |  |  |  |  |  |  |
| People who are important to me would go to the GP if a tick had been stuck on their skin for more than 24 hours. |  |  |  |  |  |  |  |  |
| People who are important to me think that it important that I go to the GP if a tick had been stuck on my skin for more than 24 hours. |  |  |  |  |  |  |  |  |

18. Imagine that you want to go to an area where there could be ticks ('green space'). Please indicate below what you would be intending to do.

*When we talk about green spaces we mean being outdoors in nature areas such as woods, heathland, dunes or a park.*

|  | 1 – strongly disagree | 2 | 3 | 4 -neutral | 5 | 6 | 7 – strongly agree |
| --- | --- | --- | --- | --- | --- | --- | --- |
| I intend to check my body for ticks after every visit to a green space. |  |  |  |  |  |  |  |
| If I find a tick on my body I intend to remove it immediately. |  |  |  |  |  |  |  |
| If I find a tick on my or someone else’s body I intend to write down the place of the bite on the body and the date. |  |  |  |  |  |  |  |
| If I have a tick on my skin for more than 24 hours I plan to go to the GP. |  |  |  |  |  |  |  |

The following questions concern the measures you take to prevent Lyme disease.

*When we talk about green spaces we mean being outdoors in nature areas such as woods, heathland, dunes or a park.*

19. How often over the past year/month(*t2*) have you checked yourself or someone else for tick bites after visiting a green space?

- 1 – never after visiting a green space
- 2
- 3
- 4
- 5
- 6
- 7 – every time after visiting a green space
- I have not visited a green space at all

20. How often over the past year/month(*t2*) have you immediately removed a tick from your own or someone else’s skin after discovering a tick bite?

- 1- never after a tick bite
- 2
- 3
- 4
- 5
- 6
- 7 – every time after a tick bite
- I have not found any tick bites over the past year/month (*t2*)

20. How often over the past/ year month (*t2*) after a tick bite have you noted down the place on the body and the date of the bite?

- 1 - never after a tick bite
- 2
- 3
- 4
- 5
- 6
- 7 – every time after a tick bite
- I have not found any tick bites over the past year

21. How often over the past year/month (*t2*) have you been to the GP with a tick that has been on the skin for longer than 24 hours?

- 1 - never after a tick bite
- 2
- 3
- 4
- 5
- 6
- 7 – every time after a tick bite
- Over the past year/month (*t2*) I have not found a tick bite that has been on the skin for longer than 24 hours.
- I go to the GP for every tick bite, even if it has been on the skin for less than 24 hours.

22. Finally, a few questions about the film/leaflet than you have seen/read.

|  | 1 – strongly disagree | 2 | 3 | 4 -neutral | 5 | 6 | 7 – strongly agree |
| --- | --- | --- | --- | --- | --- | --- | --- |
| I thought the film/leaflet was logical |  |  |  |  |  |  |  |
| I thought the film/leaflet was the right length |  |  |  |  |  |  |  |
| I feel that I understood the film/leaflet very well |  |  |  |  |  |  |  |
| I think I that would need help from someone else to understand the film/leaflet properly |  |  |  |  |  |  |  |
| I think that a lot of people would find the film/leaflet useful |  |  |  |  |  |  |  |

Your help with this study is much appreciated. To finish, we would like to know what you think of this study.
Please give your answers as if you were rating a report. 1 means a very poor report and 10 means a very good report. Of course you can give any number in between.

23. Please indicate on a scale of 1 to 10 how enjoyable or how unenjoyable you found taking part in this study

*Rate a 1 if you think that taking part in this study was not at all enjoyable and a 10 if you think it was very enjoyable.*

- 1
- 2
- 3
- 4
- 5
- 6
- 7
- 8
- 9
- 10
- Don’t know
- No report

24. You have rated this study with a/an x. Please explain in a little more detail why you rated it as such.

|  |
| --- |
