# Supplementary material for: Comparing the effect of a leaflet and a movie in preventing tick bites and Lyme disease in The Netherlands
Source: BMC Public Health. 2016 Jun 10;16:495. doi: 10.1186/s12889-016-3146-2 (PMC4902941; doi:10.1186/s12889-016-3146-2)
Supplement: Additional file 2: — Additional figures not significant results. (DOCX 133 kb) [file 12889_2016_3146_MOESM2_ESM.docx]

**Appendix 2: Additional figures not significant results**


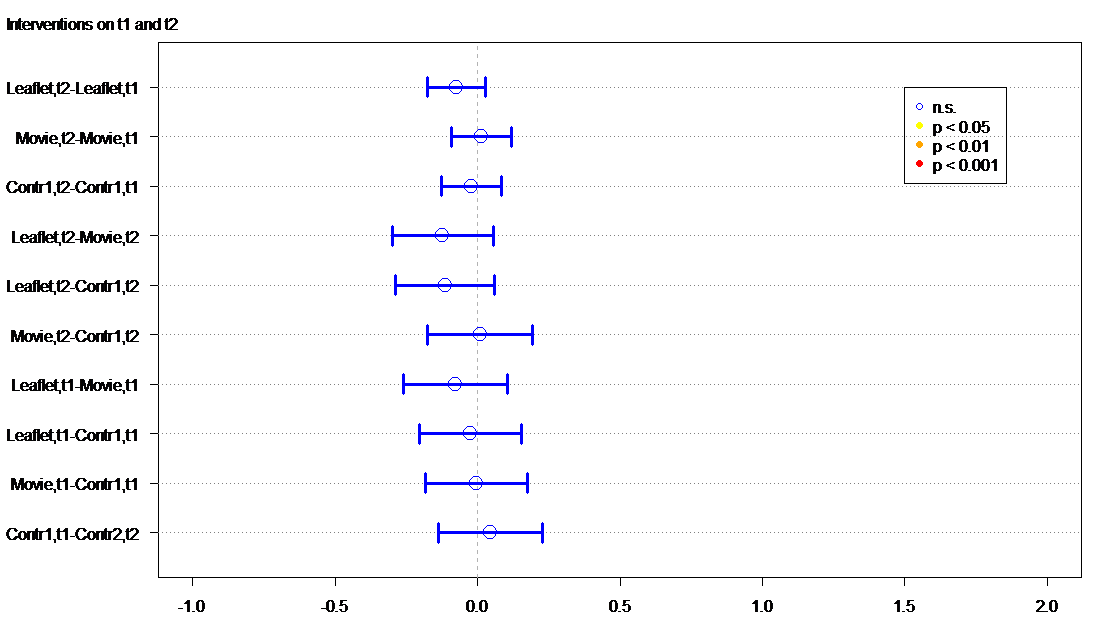


Figure 9: Differences in mean Likert scores on perceived severity overall between intervention groups


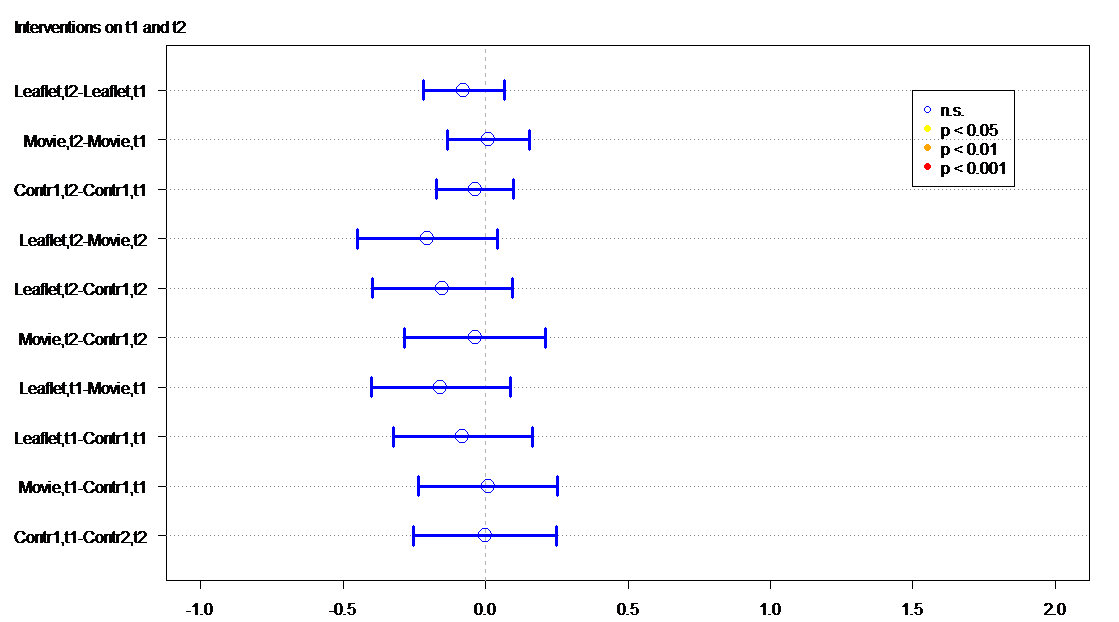


Figure 10: Differences in mean Likert scores on anxiety overall between intervention groups


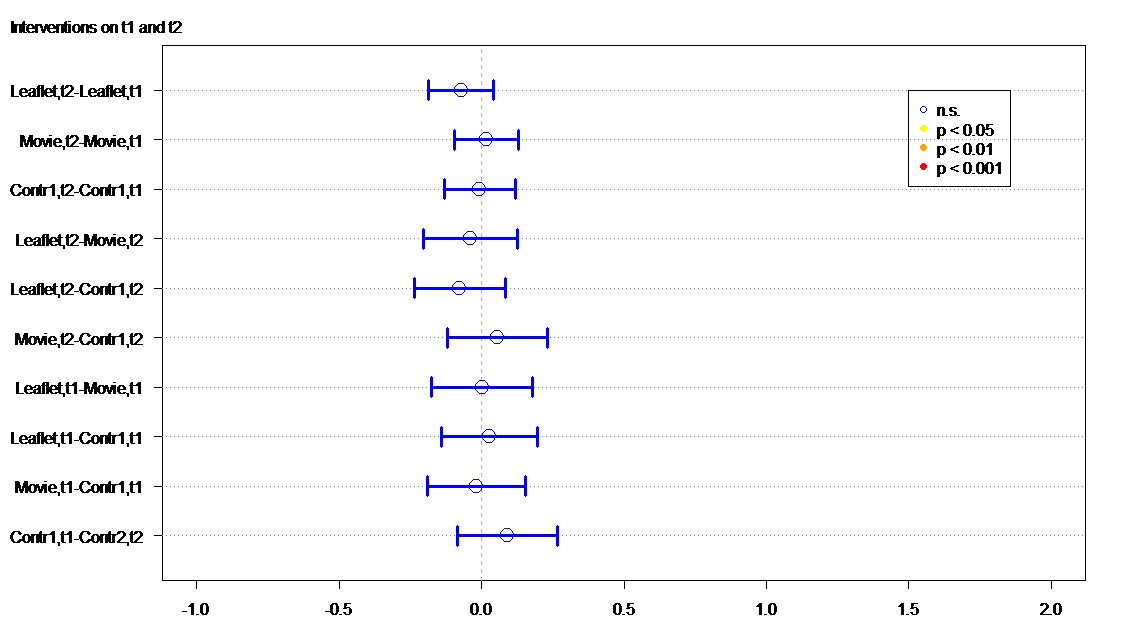


Figure 11: Differences in mean Likert scores on seriousness overall between intervention groups


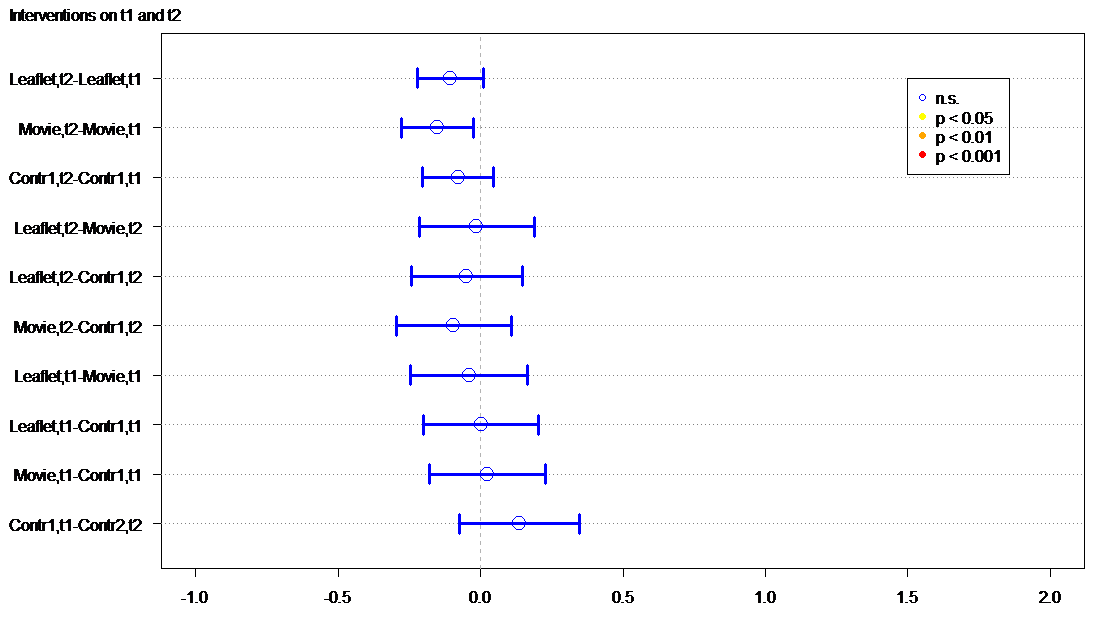


Figure 12: Differences in mean Likert scores on susceptibility overall between intervention groups
